# Overcoming Conflicting Definitions of “Euthanasia,” and of “Assisted Suicide,” Through a Value-Neutral Taxonomy of “End-Of-Life Practices”

**DOI:** 10.1007/s11673-023-10230-1

**Published:** 2023-02-02

**Authors:** Thomas D. Riisfeldt

**Affiliations:** Department of Philosophy, University of New South Wales, High St, Kensington, Sydney, New South Wales 2052 Australia

**Keywords:** Bioethics, Euthanasia and assisted suicide, Assisted dying, End-of-life issues, Informed consent, Palliative care

## Abstract

The term “euthanasia” is used in conflicting ways in the bioethical literature, as is the term “assisted suicide,” resulting in definitional confusion, ambiguities, and biases which are counterproductive to ethical and legal discourse. I aim to rectify this problem in two parts. Firstly, I explore a range of conflicting definitions and identify six disputed definitional factors, based on distinctions between (1) killing versus letting die, (2) fully intended versus partially intended versus merely foreseen deaths, (3) voluntary versus nonvoluntary versus involuntary decisions, (4) terminally ill versus non-terminally ill patients, (5) patients who are fully conscious versus those in permanent comas or persistent vegetative states, and (6) patients who are suffering versus those who are not. Secondly, I distil these factors into six “building blocks” and combine them to develop an unambiguous, value-neutral taxonomy of “end-of-life practices.” I hope that this taxonomy provides much-needed clarification and a solid foundation for future ethical and legal discourse.

Euthanasia and assisted suicide are of the utmost practical significance for many patients considering their options at the end of life, as well as for their families and health professionals involved in their care. The ongoing ethical and legal evaluation of these practices is therefore paramount. However, definitional confusion, ambiguities, and biases plague the literature and are counterproductive to this important discourse. Establishing unambiguous and agreed-upon definitions for the central terms of the euthanasia debate is essential for further fruitful ethical and legal discussion. In this essay I aim to rectify this problem. To achieve this, in section 1 I explore six aspects of the definitions of “euthanasia” and “assisted suicide” which are often disputed. In section 2 I break these down into six “building blocks” upon which I develop an inclusive, precise, and value-neutral taxonomy of what I call the “end-of-life practices.” I hope that this taxonomy leads to a greater appreciation of the range of different practices at the end of life and that it reveals some of the hidden metaphysical challenges underlying many of these distinctions. Although the taxonomy is strictly and deliberately value-neutral, I believe that it provides much-needed clarification and a solid foundation for future ethical and legal discourse. In particular, I envisage that readers may utilize it as a platform for their ethical arguments—particularly by way of comparing the ethical significance of the similarities and differences between the various delineated practices—and that it may also aid policymaking and the design of empirical studies. It should also be noted that “euthanasia” may apply to non-human animals (in the veterinary context) as well as to humans, although this article focuses exclusively on humans.

## Section 1. There Are No Consensual Definitions of “Euthanasia” or “Assisted Suicide” Owing to Six Disputed Definitional Factors

### Paradigm Cases

“Euthanasia” is etymologically derived from the Greek for “good death,” and there is widespread agreement amongst interlocutors that certain *paradigm cases* constitute euthanasia or assisted suicide respectively; this forms a good starting point for discussion. Suppose that a man named “X” has end-stage metastatic bowel cancer and is slowly dying.^1^ He is fully conscious and does not have dementia or any other form of cognitive impairment. He is suffering from the physical symptoms of pain, shortness of breath, nausea, and vomiting. He is largely bedbound and has lost most of his independence, including the capacity to toilet independently. He has become weary of life; he no longer enjoys many activities or has meaningful life pursuits. He does not want to continue being an emotional and financial burden on his family. Despite this, he is not clinically depressed and does not feel in any way coerced by his family. X decides that he wants to control the manner and timing of his death. For all of these reasons he asks you, his physician (“doctor”), to kill him by administering a lethal injection of a sedative/anaesthetic drug called pentobarbital. You comply, with the full intention of killing him, as a means to relieve his suffering. This is a paradigm case of euthanasia. Suppose that X instead asks you to provide him with a lethal combination of oral drugs which he can take himself at home. Again you comply, with the full intention of those drugs killing him, as a means to relieve his suffering. This is now a paradigm case of assisted suicide. The term “assisted dying” will be discussed later.

### Methodology for Identifying Disputed Definitional Factors

Although there is widespread agreement amongst interlocutors that certain paradigm cases like the ones offered above constitute euthanasia or assisted suicide, there is rampant disagreement as to what the correct definitions of the terms actually are and whether or not particular *non-paradigm cases* constitute euthanasia or assisted suicide. Definitions can be sought from various medical and ethical organizations, from parliamentary legislature and common law, from empirical studies, and from independent bioethicists and other authors writing in the field. These medical and ethical organizations and laws exist at global, continental, national, and state/territory/provincial levels, and medical bodies exist as national medical associations and also as subspecialty associations (particularly adult internal medicine and palliative care subspecialities). This results in quite literally hundreds of definitions of euthanasia and assisted suicide, if not more. To complicate matters further, these definitions have been dynamic over time; they have existed for centuries and have been rapidly evolving, particularly in the last few decades, and it must be acknowledged that there has been a degree of convergence amongst certain interlocutors in recent years. A methodological approach of analysing hundreds of individual definitions is unfeasible, and selecting a representative sample of these definitions without being ad hoc and introducing a selection bias is a challenging task. In the first part of this essay I approach this challenge by selecting a range of definitions spanning the aforementioned types of sources, across multiple continents, and spanning parties who are proponents and opponents of euthanasia and assisted suicide, in an attempt to minimize any such bias. In any case, all that is required for the thesis that follows is to review a sufficient number and type of definitions, in order to identify the various disputed definitional factors that then warrant further evaluation. In the meantime I will focus on definitions of euthanasia and will turn to assisted suicide thereafter.

### Global and National Definitions of Euthanasia

To begin at a global level, the World Health Organization has recently retracted its definition and any official position on euthanasia. The next largest global medical body, the World Medical Association (WMA 2015, 59), most recently defines euthanasia as:

[…] knowingly and intentionally performing an act that is clearly intended to end another person’s life and that includes the following elements: the subject is a competent, informed person with an incurable illness who has voluntarily asked for his or her life to be ended; the agent knows about the person’s condition and desire to die, and commits the act with the primary intention of ending the life of that person; and the act is undertaken with compassion and without personal gain. […] Euthanasia and assisted suicide, according to these definitions, are to be distinguished from the withholding or withdrawal of inappropriate, futile or unwanted medical treatment or the provision of compassionate palliative care, even when these practices shorten life.

Now turning to national medical organizations, the British Medical Association (BMA 2021, ¶8, 10) defines “voluntary euthanasia” as being where “doctors would administer lethal drugs at the voluntary request of an adult patient with capacity, who meets defined eligibility criteria, with the intention of ending that patient’s life,” and it is restricted to patients that “have either a terminal illness or serious physical illness causing intolerable suffering that cannot be relieved.” The American Medical Association (2016, 10) most recently defines euthanasia as “the administration of a lethal agent by another person to a patient for the purpose of relieving the patient’s intolerable and incurable suffering.” The Australian Medical Association (2016, 1–2) most recently defines euthanasia as “the act of deliberately ending the life of a patient for the purpose of ending intolerable pain and/or suffering,” and specifically excludes from their definition any instance of “not [initiating] or continuing life-prolonging measures, or the administration of treatment or other actions intended to relieve symptoms which may have a secondary consequence of hastening death.”

Now let us turn to definitions arising from empirical studies from the Netherlands and the legislature resulting from these. Van der Maas, van Delden, and Pijnenborg (1992, 23), in the English-version account of the “Commission of Inquiry into the medical practice concerning euthanasia” (aka the “first Remmelink Report”), define euthanasia as “the purposeful acting to terminate life by a person other than the person concerned upon request of the latter.” Related literature in this field, notably van der Maas et al. (1991), van der Wal and Dillmann (1994), van der Maas et al. (1996), Onwuteaka-Philipsen et al. (2003), and van der Heide et al. (2007) all define euthanasia in similar ways. Legemaate (2004), which constitutes the closest to an “official” English translation of the Netherlands’s “Termination of Life on Request and Assisted Suicide (Review Procedures) Act 2002,” also uses the term similarly; this is testament to the considerable efforts to maintain definitional consistency amongst this group of Dutch authors. Legal literature from other European countries such as Belgium, Luxembourg, Switzerland, and Germany could equally have been reviewed here. I will now utilize the definitions just stated as a starting point to explore the disputed definitional factors found across the literature as a whole, again focusing on euthanasia for the time being.

It should first be noted that there are six challenges mentioned herein for which any detailed examination lies beyond the scope of this essay. Three of these challenges are epistemological, regarding the practical difficulties in determining the provider’s true intentions, in determining whether informed consent has truly been provided by the patient, and in determining whether an illness is actually terminal, all on an individual/case-by-case basis. Two of these challenges are metaphysical, regarding the definition of death and regarding theories of causation in the context of two simultaneous causally relevant factors (i.e., simultaneous actions and omissions). The last challenge is empirical, pertaining to whether or not palliative opioid and sedative use hasten or bring about death (along with how this relates to Aquinas’s doctrine of double effect). These are highlighted again in the relevant sections below.

1. Is Euthanasia Necessarily a Killing or Can It Also Be a Letting Die?

Firstly, I need to distinguish “killing” from “letting die.” Most interlocutors agree that killing is an action or commission, as opposed to letting die which is an omission. Killing in the medical context is usually achieved through injecting a lethal bolus (a large single dose) of a drug. This drug may have analgesic and/or sedative properties (e.g., morphine, an opioid; or pentobarbital, a barbiturate), or it may not (e.g., potassium chloride, an ionic compound which in high doses stops the heart from beating). A combination of drugs may be used. Letting die in the medical context occurs through “withholding or withdrawing inappropriate or futile life-sustaining treatments” (WWIFLSTs). I have chosen this rather cumbersome phrase because it precisely encapsulates the terminology used in the literature.

“Medical futility” is a complex and often controversial concept (Emanuel 2018; Fins, McCarthy, and Limehouse 2017; Materstvedt and Bosshard 2009). For the purpose of this essay it should suffice that a “futile treatment” is one that strives for a medical benefit despite that benefit being impossible to achieve. A “life-sustaining treatment” is any treatment that prolongs life without reversing the underlying medical condition. The prototypical example of *withholding* life-sustaining treatment is the Do Not Resuscitate (DNR) order (Fins, McCarthy, and Limehouse 2017). More than 98 per cent of patients in intensive care units die without cardiopulmonary resuscitation being attempted (Emanuel 2018), many of whom would have had DNR orders in place. Another example of withholding life-sustaining treatment is electing not to treat bacterial chest infections with antibiotics. Examples of *withdrawing* life-sustaining treatments are turning off mechanical ventilators or dialysis machines. Turning off implanted cardiac devices (e.g., permanent pacemakers) constitutes withholding life-sustaining treatment if the device only has a stand-by function or withdrawing life-sustaining treatment if the device has a continuous function (Fins, McCarthy, and Limehouse 2017). On average, 3.8 life-sustaining treatments are withheld or withdrawn per dying intensive care unit patient (Emanuel 2018). Artificial nutrition and hydration are usually considered to be life-sustaining treatments which can be withheld or withdrawn like any other, although this is controversial (American Academy of Hospice and Palliative Medicine AAHPM 2011, 2013; Jonsen, Siegler, and Winslade 2010).^2^

The distinction of killing versus letting die is important because it reflects the dichotomy between “active” and “passive” euthanasia used by some interlocutors; this is synonymous with “direct” and “indirect” euthanasia, respectively. On this account, “active euthanasia” is where the person being euthanized (the patient) is *killed* by the person providing the euthanasia (usually a doctor). Conversely, “passive euthanasia” is where the doctor *lets the patient die* by WWIFLSTs, thereby failing to prevent or delay death “from natural causes” (the underlying disease) where the means for doing so was available. This dichotomy is central in Rachels (1975) and is also recognized by Kerridge, Lowe, and Stewart (2013), whereas it is rejected by the WMA, BMA, American Medical Association, Australian Medical Association, and the authors from the Netherlands, all of whom state that euthanasia is necessarily an “act.” This position is also shared by the AAHPM (201), Australian and New Zealand Society of Palliative Medicine (ANZSPM 2013), Karlawish and James (2009), Materstvedt and Bosshard (2009), and Singer (2011). On this definition the qualifier “active” is superfluous and “active euthanasia” is tautological; conversely, “passive euthanasia” is a contradiction in terms. The distinction is confused further by virtue of some interlocutors stating that “killing through omission” exists or that the metaphysical distinction between killing and letting die rests on an untenable theory of causation altogether. For example, Warnock (1998, 32) claims that “I can kill my pet by neglect as well as by running her over,” and Harris (1975, 83) claims that “if a doctor refuses to treat a patient [when the means for treating that patient were readily available], he has killed that patient as sure as shooting.” Furthermore, some authors use the terms just discussed inconsistently, such as by defining “active euthanasia” as a form of killing and “passive euthanasia” as a form of WWIFLSTs but then claiming that WWIFLSTs is separate from and does not constitute euthanasia (Emanuel 2018).

2. Is Euthanasia Necessarily Fully Intended (On Behalf of the Provider) Or Can It Also Be Partially Intended?

The WMA, BMA, American Medical Association, Australian Medical Association, and the authors from the Netherlands all agree that euthanasia necessarily intends to hasten or bring about death, as opposed to merely foreseeing (but not intending) the hastening or bringing about of death. This is important in the context of two palliative care practices: the appropriately titrated administration of opioids and continuous deep palliative sedation, which—controversially—may hasten or bring about death. Supposing that they do, these two practices are distinct from euthanasia on this orthodox definition provided that they merely foresee (but do not intend) that death will be hastened or brought about. Arguments as to whether or not either of these practices actually do hasten or bring about death, along with arguments regarding their applicability to Aquinas’s doctrine of double effect and arguments regarding the validity of the doctrine itself (McIntyre 2019), all lie beyond the scope of this essay.

This distinction is complicated further because the intention for death to be hastened or brought about is not always simply present or absent; a person can have simultaneous intentions, and those intentions can be weighted differently. The contentious question remains as to whether the intention to hasten or bring about death needs to be the *only* intention present or even the *main* intention present, for a practice to constitute euthanasia. The WMA and the authors from the Netherlands qualify that the intention has to be “primary” or “explicit” (which can also be considered synonymous with a “full,” “main,” or “principal” intention); however, the other definitions referenced thus far do not use this qualification. Furthermore, hastening or bringing about death can also be “partially” or “secondarily” intended, or at least “not unwelcomed” (these terms are all synonymous), as opposed to being absent altogether (and the death therefore being completely “unintended” or “merely foreseen”; these terms are also synonymous). According to the definitions from the WMA and the Dutch literature, partially intended killing does not constitute euthanasia because it is not fully intended. Conversely, most of the other aforementioned sources disagree, claiming that euthanasia extends to killing with *any* intention of bringing about or hastening death, irrespective of whether that intention is full or partial. The epistemological challenge of ever actually knowing what another person’s true intentions are in this context is a valid concern which falls beyond the scope of this essay; however, it does not undermine the metaphysical importance that intention has in this context. Any exploration of the definition of euthanasia without reference to the intention of the provider would be incomplete.

3. Is Euthanasia Necessarily Voluntary (On Behalf of the Patient) Or Can It Also Be Nonvoluntary or Involuntary?

The definitions from the WMA and the Dutch literature all state that euthanasia is necessarily voluntary. The American Medical Association and Australian Medical Association do not explicitly state this, although it could be considered implied through their discussions being in the context of patient requests for euthanasia. The ANZSPM (2013) and Materstvedt and Bosshard (2009) also share this view, upon which the qualifier “voluntary” is superfluous, “voluntary euthanasia” is tautological, and “nonvoluntary euthanasia” and “involuntary euthanasia” are contradictions in terms. Note that the BMA defines “voluntary euthanasia” as being necessarily voluntary, although I wonder whether this tautological statement is deliberate so as to emphasize the importance of voluntariness. Conversely, some other authors reject the claim that euthanasia is necessarily voluntary (e.g., Emanuel 2018).

The terms “voluntary,” “nonvoluntary,” and “involuntary” refer to the “informed consent” provided or not provided by the patient. The bioethical and legal understandings of informed consent are closely intertwined; most interlocutors agree that at the most basic level there are three necessary and sufficient criteria for informed consent: sufficient provision of information, sufficient decision-making capacity (aka “medical competency”), and freedom from coercion (see Eyal 2019, ¶2 under “The requirement of informed consent”; Hawkins and Charland 2020). Some authors may subdivide these same features into more than three parts, although the way that I have stated them here is sufficient to define the concept of informed consent. If a patient provides informed consent to a practice then it is voluntary and if he *expressly does not* consent to a practice then it is involuntary; whereas if he neither consents to nor expressly does not consent to a practice then it is nonvoluntary.^3^ An example of this outside of the context of euthanasia would be a patient presenting with fevers and neck stiffness who, from the doctor’s perspective, requires a lumbar puncture (spinal tap) to evaluate for possible meningitis. If the doctor offers this procedure to the patient and the patient consents to it, then the procedure is done voluntarily; if the patient says that he does not want the procedure and the doctor goes ahead and performs it regardless, then the procedure is done involuntarily; and if the patient is unconscious because they are critically unwell with meningitis and they are therefore unable to say either yes or no to the procedure, and the doctor then goes ahead with the procedure, then it is done nonvoluntarily. In the context of euthanasia this trichotomy may be adopted (e.g., Young 2021), or it may be rejected such as by adopting a dichotomy instead; Karlawish and James (2009) do this by distinguishing “competent” from “noncompetent” euthanasia. The epistemological challenge of distinguishing voluntary, nonvoluntary, and involuntary practices on a case-by-case basis in the context of euthanasia is also a valid concern which falls beyond the scope of this essay; although again it does not undermine the metaphysical importance of voluntariness in this context, and any exploration of the definition euthanasia without reference to voluntariness would be incomplete.

4. Is Euthanasia Necessarily Provided to Patients Who Are Terminally Ill or Can It Also Be Provided to Patients Who Are Not Terminally Ill?

The WMA’s definition states that euthanasia is necessarily provided to patients who are terminally ill, whilst the BMA’s definition states that it necessarily applies to terminally ill patients *or* those who have a “serious physical illness causing intolerable suffering that cannot be relieved.” Definitions from the American Medical Association, Australian Medical Association, and the Dutch literature do not explicitly state that euthanasia is necessarily provided to terminally ill patients, although they are all discussed in this context. Karlawish and James (2009) state that euthanasia is necessarily provided to terminally ill patients *or* those in permanent comas (more on this shortly). Conversely, some interlocutors argue that euthanasia can also include patients who are not terminally ill (see the Royal Dutch Medical Association KNMG 2011 and Young 2021 for further discussions on this point).

A “terminal illness” is an incurable illness which will inevitably result in death unless something else—e.g., an acute traumatic injury—does first. End-stage metastatic bowel cancer is prototypical of a terminal illness, whilst an isolated chest infection (“pneumonia”) which is acutely treatable and fully reversible in a particular patient is prototypical of a non-terminal illness. However, it is important to recognize that there is a significant grey area here. Firstly, an illness which will inevitably result in death might be incurable because a cure is just not *available,* e.g., end-stage kidney disease is sometimes curable with a kidney transplant but transplants are often unavailable. Secondly, an illness which is curable might not have an *acceptable* cure, e.g., a difficult course of surgery, chemotherapy, and radiotherapy for a cancer may be unacceptable for some patients. Thirdly, an illness might have been curable at some point in the past but is no longer curable, e.g., cancer which has spread throughout the body after electing not to treat it earlier on when it was more anatomically localized. Fourthly, a collection of illnesses might together be incurable, e.g., although chronic conditions such as obesity, hypertension, diabetes, and some forms of heart and lung disease; acute conditions such as broken hips and chest infections; and complications such as being immobile and bed-bound, are not terminal illnesses on their own, a patient with all of these conditions and complications simultaneously might not be curable in any practical sense. Fifthly, terminal illnesses form a heterogeneous group; someone might live for up to twenty-five years with a very slowly-progressing variant of Alzheimer’s disease, whereas another person might only live for a few months with a very aggressive cancer. However, it is worth noting that some interlocutors reject this orthodox claim and define a terminal illness with some form of time constraint, e.g., Battin et al. (2007) use a “prognosis less than six months” criterion. Finally, an illness which is currently incurable might become curable in the future, e.g., if a cure for Alzheimer’s disease is achieved. It might even be pessimistically and cynically claimed that, in lieu of a means to immortality, life itself fits the definition of a terminal illness. Distinguishing between terminal and non-terminal illnesses on a case-by-case basis in practice is another epistemological problem altogether which falls beyond the scope of this essay; although again it does not undermine the metaphysical importance of terminal illness in this context, and any exploration of the definition of euthanasia without reference to terminal illness would be incomplete.

5. Is Euthanasia Necessarily Provided to Patients Who Are Fully Conscious or Can It Also Be Provided to Patients Who Are in Permanent Comas or Persistent Vegetative States?

Another disputed factor is whether or not euthanasia applies to patients in permanent comas or persistent vegetative states (PVSs). In permanent comas, patients are completely unconscious. They lack sleep-wake cycles and cannot be woken, fail to respond normally to painful stimuli along with light and sound, and cannot initiate voluntary movements. The qualifier “permanent” is contentious since it implies diagnostic certainty which is inconsistent with a few rare but well-documented cases of patients “waking up” after being thought to be in permanent comas (Jonsen, Siegler, and Winslade 2010). Given that the vast majority of patients thought to be in permanent comas really are in permanent comas, and that diagnostic uncertainty features in the diagnosis of almost every medical condition, I will abstract from this uncertainty and accept the qualifier “permanent” for the purpose of this essay. PVSs are similar, although patients do have sleep-wake cycles and are partially rousable by painful stimuli, light, and sound. However, they completely lack awareness of themselves and their external environments and cannot interact with their environments in any way or initiate voluntary movements (Jonsen, Siegler, and Winslade 2010). Permanent comas and PVSs may arise from acute events, e.g., traumatic head injuries or going five minutes without oxygen in a near-drowning, or may result from the end stages of a progressive neurological decline, e.g., in Alzheimer’s disease.

None of the definitions from the WMA, BMA, American Medical Association, Australian Medical Association, or the Dutch literature specifically state whether or not euthanasia applies to patients in permanent comas or PVSs, although they do all state that an expressed autonomous decision for euthanasia is necessary. Therefore, euthanasia would have to be provided to a patient who is fully conscious if that decision were to be made contemporaneously, although euthanasia could possibly also apply to a patient in a permanent coma or PVS if this autonomous decision was expressed pre-emptively via an advanced care directive (ACD). Note that Singer (2011) and van der Wal and Dillmann (1994) explicitly exclude comatose patients from the definitions of euthanasia; Singer argues that this is because euthanasia is by definition for the purpose of relieving suffering, whereas patients in permanent comas are incapable of experiencing pain or suffering.

Definitions of death are also critical here. According to the “whole brain” (or “brainstem”) definition of death which is almost unanimously accepted in legal jurisdictions and in clinical medicine globally, patients in permanent comas or PVSs are living persons. On this definition, “death” is “the irreversible cessation of functioning of the entire brain, including the brainstem” (DeGrazia 2021, ¶1 under “The Current Mainstream View”), and patients in permanent comas or PVSs *do* have brainstem function. However, there is a competing “higher brain” definition of death, whereby death is “the irreversible cessation of the capacity for consciousness” (DeGrazia 2021, ¶1 under “A Progressive Alternative”). On this definition, patients in permanent comas or PVSs are not living persons because they lack consciousness or the capacity for future consciousness, despite the fact that the part of their brain which controls respiration and cardiac function (the brainstem) is still functioning. Consequently, if this “higher brain” definition is correct and patients in permanent comas or PVSs are not actually living persons, then euthanasia cannot apply to them. Arguments for and against these competing definitions of death fall beyond the scope of this essay, although I will adopt the more orthodox whole brain definition here for the purpose of taxonomic inclusiveness.

6. Is Euthanasia Necessarily Provided to Patients Who Are Suffering or Can It Also Be Provided to Patients Who Are Not Suffering?

Stemming from its etymological roots, euthanasia is usually viewed as necessarily being provided to patients who are suffering, in a compassionate way, since this is the very purpose of euthanasia in the first place. It is usually argued that without suffering there should be no reason for a patient to consider euthanasia in the first place, so any conceptualization of euthanasia in this context would be misplaced. The BMA, American Medical Association, and Australian Medical Association all define euthanasia as necessarily being provided to patients who are suffering, using the qualifier “intolerable” on each occasion. The WMA does not explicitly state this, although it could possibly be implied from the necessity that euthanasia is “performed with compassion.”

It should be noted that the presence or absence of suffering in the context of euthanasia is also significant because the bioethical principles of beneficence and non-maleficence are largely dependent upon mitigating or not causing suffering, respectively. These principles may in turn be based upon concepts of natural law, derived from either secular or theistic origins. Bioethical arguments may then appeal to these principles and to concepts of natural law to argue for euthanasia or to argue against euthanasia (e.g., Pellegrino 2005). However, further discussion of these arguments would involve value judgements which do not fall within the value-neutral task of this essay.

Furthermore, the presence or absence of suffering is intricately related to the fourth question just discussed concerning terminal illness. Firstly, let us consider patients who are terminally ill. Most of these patients will have some form of physical suffering, e.g., symptoms of pain, shortness of breath, or nausea or vomiting, such as in the settings of advanced cancers or the end stages of chronic heart, lung, kidney, or liver failure. Most will also experience at least some form of non-physical suffering, e.g., emotional/psychological or existential suffering. One possible caveat to this is the case of dementia (of which Alzheimer’s disease is a subtype). In this disease—which is essentially the end-stage of chronic brain failure to continue the above analogy—there may conceivably be instances in which the patient does not have physical symptoms and also does not experience any non-physical suffering (owing to complete oblivion regarding themselves, their diagnosis, and their environment).

The question of just *how much* suffering, or just *how unrelievable* that suffering has to be, is also problematic. Suffering clearly exists on a spectrum from mild to intolerable, and the extent to which suffering can be relieved also falls on a spectrum from complete relief (e.g., an antiemetic for mild nausea) to minimal relief (e.g., analgesia for a treatment-refractory severe headache or chronic pain syndrome). Drawing a concrete line somewhere along each of these spectrums is likely to be arbitrary, which is metaphysically troublesome. However, it would seem that a line *does* need to be drawn at some point, because the trivial suffering at one end of the spectrum (e.g., a sore finger caught in a car door) clearly does not meet the suffering criterion stipulated by most authors as being necessary for euthanasia. Furthermore, the extent of suffering needs to be determined by the person enduring it (as opposed to the outsider observing it), which can pose an additional challenge in the unconscious patient who is unable to communicate; however, there are often objective signs, such as groaning or wincing, that are evidence of suffering in these patients. There is also the further question as to whether non-physical suffering in the absence of physical suffering can ever be sufficient to meet the “intolerable suffering” threshold posited by many interlocutors.

Focusing now on patients who are not terminally ill, there will nonetheless usually be suffering present as the cause for the request for euthanasia in the first place. This is particularly the case if at least some degree of suffering, rather than intolerable and unrelievable suffering, is being considered. The main caveat to this is the case of non-terminally ill people who are simply “done with life,” otherwise stated as “having completed life” or possibly “being tired of life.” The terminology here is challenging; to me, it intuitively refers to someone who does not have any further uncompleted life goals and is ready to not be alive anymore. Existential beliefs are crucially important here; religious or spiritual beliefs in an afterlife may circumvent existential angst and ultimately lead to a mindset of feeling ready to “pass on to the next life,” whilst at the same time atheists might not experience existential angst and instead feel at peace with, and be ready to confront, this great “finality.” Furthermore, if a person is truly not suffering, then calling them a “patient” may be inappropriate and should perhaps be replaced with “recipient,” although I will continue to use “patient” in this essay.

However, contrary to my own intuitions, most interlocutors referring to people who have “completed” or “are done with” life are actually referring to a state of suffering in non-terminally ill patients. The Royal Dutch Medical Association (KNMG 2011, 14) certainly does not advocate for the term “euthanasia” applying in this context, although it does offer a description of the theory, explaining that:

According to Right to Die-NL (NVVE) the concept of a “completed life” is used for “people who suffer from a complex constellation of factors connected with old age. These are non-life-threatening conditions and physical deterioration (poor eyesight, deafness, difficulty walking, fatigue, apathy, incontinence), resulting in a loss of independence and personal dignity, dependence on care, loss of status and control, a shrinking social network, loss of a sense of purpose and meaning, disengagement from society, fear of the future and the absence of future prospects”

The presence or absence of suffering is also intricately related to our third question discussed earlier. I think that it is contentious as to whether a desire for euthanasia in the absence of significant suffering can ever be truly voluntary, or whether this is only possible in the context of depression or another mental illness, thereby undermining decision-making capacity and hence voluntariness to some extent. The concept of euthanasia provided to people who are not suffering (or at least not suffering from intolerable and unrelievable suffering in the context of a terminal illness) is almost invariably posited in the context of elderly patients. A reason for this is likely because of an assumption that these thoughts in a young person would *necessarily* be in the context of mental illness. This introduces yet another metaphysical challenge in drawing a non-arbitrary line upon another spectrum: how old is old enough? In the absence of suffering, is a ninety-year-old allowed to be “done with life” without necessarily suffering from a mental illness, whereas a forty-year-old would necessarily be depressed if he shared this belief? What about a sixty- or an eighty-year-old?

The presence or absence of suffering is also intricately related to our fifth question discussed earlier. As previously highlighted, it is contentious as to whether or not patients in permanent comas and PVSs can experience suffering. Given that patients in permanent comas and PVSs are, by definition, not consciously aware of themselves or their surrounding environments, it could be argued that they are physically incapable of suffering because suffering is a conscious, lived experience. However, it could also be argued that an outside observer cannot ever really know what the first-hand experience of being in a permanent coma or PVS would be like, à la Nagel’s “What is it like to be a bat?” (1974); hence, we may never know if these patients are capable of experiencing suffering, and this may even be a non-falsifiable claim. The distinction between permanent comas and PVSs is also potentially relevant here, given that only the latter respond to painful stimuli; this might be claimed as evidence of suffering, or conversely it might just be considered to be an unconscious, reflexive motor response to a noxious stimulus. As a separate notion altogether, it might be argued that patients in permanent comas and PVSs *must* be suffering, based on an evaluation of their quality of life, although this is a value judgement from the perspective of an outsider which may cast doubt on the tenability of this position. To complicate matters further, if the patient in the permanent coma or PVS is not really a “person” at all on the higher brain definition of death, then any discussion on the presence or absence of suffering experienced *by a person* becomes moot in this setting. Although I will not take a position on any of these arguments within the scope of this essay, they do importantly demonstrate that *if* persons in permanent comas or PVSs are incapable of suffering, and *if* euthanasia extends to persons in permanent comas and PVSs, then it follows that suffering is *not* a necessary criterion for a practice to constitute euthanasia. Singer’s (2011) focus on suffering as a necessary feature of euthanasia, and his excluding patients in permanent comas from the definition of euthanasia, may be partly or entirely owing to this.

### Definitions of “Assisted Suicide” and “Assisted Dying”

When sources define “euthanasia” as being necessarily an act, they define “assisted suicide” in the same way, except that in assisted suicide the second party provides the information and/or means for the patient to commit suicide, rather than killing the patient himself. Because assisted suicide requires the (active) provision of information or means, there cannot be “passive” assisted suicide. A possible exception to this would be if a suicidal person was actively seeking out the information or means to commit suicide and the second party had the opportunity to prevent this process but decided not to, which would therefore be “passive” in a sense, although I consider this too farfetched—at least in the context of end-of-life decision-making—to warrant further discussion. The vast majority of interlocutors define euthanasia and assisted suicide as being mutually exclusive, although this is not universal; Jonsen, Siegler and Winslade (2010) and Singer (2011) suggest that assisted suicide is a subset of euthanasia, and Cholbi (2021) suggests that euthanasia is a subset of assisted suicide. The term “assisted dying” is problematic in that it is sometimes used as being synonymous with assisted suicide and is sometimes used as denoting the set containing both euthanasia and assisted suicide. It is occasionally also used in a third way as an attempt to move away from the word “suicide,” which may be considered value-tainted in a similar way to how “killing” may be considered value-tainted. This third usage aims to distinguish the practice of “assisted dying” (in the context of end-of-life decision-making) from assisted suicide outside of this context (e.g., assisting a physically healthy but depressed twenty-year-old to commit suicide).

### Previous Work Analysing the Definitional Components of “Euthanasia”

The main previous work analysing the definitional components of euthanasia that I am aware of is that of Beauchamp and Davidson (1979). This needs to be evaluated in its historical context and the landscape of the ethical and legal literature on euthanasia at that time, and represents a comprehensive and substantial effort to clearly define euthanasia and assisted suicide in a value-neutral way, in doing so recognizing the practical significance of this for both ethical and legal discussions. To summarize, Beauchamp and Davidson argue that there are five criteria which are necessary and sufficient for the definition of euthanasia: (1) the provider intends for the recipient to die and the provider causes or is causally relevant in the recipient’s death, whether it be via active or passive means, (2) the provider believes that the recipient is suffering or in a permanent coma and this belief is grounded on sufficient evidence, (3) the provider’s primary intention is to relieve the recipient’s present or predicted future suffering or irreversible comatoseness and the means by which this is achieved involves less suffering than would otherwise be present, (4) the means by which the recipient’s death is achieved is as painless as possible, unless there is an overwhelming reason to the contrary, and (5) the recipient is not a fetus (so as to separate euthanasia from abortion).^4^

There is some overlap between this definition and our own preceding discussion of the various disputed definitional factors. Regarding our first and third identified questions, Beauchamp and Davidson acknowledge that interlocutors at the time spoke in terms of the dichotomies of active and passive euthanasia, and voluntary and involuntary euthanasia, and argue that any definition of euthanasia must therefore accommodate both of these dichotomies; any other approach would be inadequate. Regarding our second question, they argue that euthanasia must be primarily intended by the provider, although that need not be the only intention. Regarding our fourth question, they argue that patients being euthanized do not necessarily have to be terminally ill. They also argue that euthanasia and “murder” are not mutually exclusive sets (some events may constitute both euthanasia and murder), which also reflects the backdrop wherein under all existing laws at the time, all instances of euthanasia constituted homicide. They then acknowledge that the term “murder” is value-loaded, whilst at the same time acknowledging that *at least some* instances of euthanasia may be unethical, so this attributed negative value might not always be misplaced. Regarding our fifth question, they state that euthanasia extends to patients in permanent comas.

Beachamp and Davison’s argument pertaining to our sixth question is particularly insightful. They argue that any definition of euthanasia *does* need to be consistent with its etymological roots of being a “good death.” To qualify as a “good death,” the patient’s death must be a better outcome (or state of affairs) than the patient still being alive. Death could be a better outcome than being alive for two reasons: either the relief of suffering or by virtue of respecting the patient’s autonomously expressed desire to die, although the relief of suffering itself is an independently necessary criterion for euthanasia. However, this can be *current* suffering or *anticipated future* suffering, e.g.*,* the symptoms of Huntington’s disease being expected to manifest twenty years after the time that the genetic diagnosis is originally made (via blood tests in an asymptomatic patient with a family history of the condition). Whilst this “anticipated future suffering” condition appears counterintuitive to me, Beachamp and Davidson then borrow an example from Hare (1975), wherein someone is trapped under a petrol lorry in a motor vehicle accident and is not currently suffering but will certainly be roasted to death in ten minutes; they argue that killing that person prior to the roasting event occurring would still constitute euthanasia. This also seems intuitive and is thus paradoxical. It suggests yet another spectrum spanning from the patient currently suffering (at time zero) to the patient suffering at some future time point (perhaps minutes or decades from now); drawing a non-arbitrary line upon this spectrum is again metaphysically troublesome.

Beauchamp and Davidson’s (1979) argument also relates to some further terminological issues. They classify assisted suicide as a subtype of euthanasia, distinguishing it from “unassisted suicide” which is not a form of euthanasia. They also argue that the word “kill” is irrecoverably value-tainted and therefore unsuitable for the definition of euthanasia, so they avoid the word altogether and replace it with “causing death.” Finally, they argue that whilst euthanasia is sometimes used synonymously with “good death” (reflecting its etymological roots), “good death” here is being used as a value-neutral placeholder only, and it does not imply that euthanasia is necessarily “good” in the sense of being necessarily ethical. Therefore, statements such as “euthanasia is bad” (which could be translated as “good death is bad”) are not contradictory, so “euthanasia” can still be used as a value-neutral (rather than a value-loaded) term.

### This Rampant Definitional Disagreement Is Problematic

The definitional disagreement evidenced in this essay thus far is problematic in a number of ways. It is confusing in clinical communication, e.g., when doctors are explaining end-of-life options to patients and their families (Materstvedt and Bosshard 2009). It is confusing when attempting to interpret and compare evidence from empirical studies investigating attitudes towards, and practices of, euthanasia in countries where it is legal or illegal (Materstvedt and Bosshard 2009). It is counterproductive to bioethical discourse; interlocutors may unwittingly be talking past each other and arguing about different practices, and linguistic disputes often distract from, or are conflated with, substantive ethical debate. It may reflect underlying ethical biases; proponents of euthanasia (e.g., Rachels 1975) may define “euthanasia” liberally, which suggests that currently accepted medical practices in palliative care already constitute forms of euthanasia, whereas opponents of euthanasia (particularly from medical and subspecialty palliative care bodies) typically do the opposite and define “euthanasia” narrowly, which stresses the differences between euthanasia and the end-of-life practices which currently fall within the scope of mainstream palliative care services. Finally, the most serious danger of all is that different practices which happen to fall under one name might erroneously be assigned the same ethical or legal statuses.

## Section 2. The Six Disputed Definitional Factors Can Be Combined to Develop a Value-Neutral Taxonomy of “End-Of-Life Practices”

### Previous Work in Delineating the Range of End-Of-Life Practices

Having established that the terms “euthanasia” and “assisted suicide” are widely disputed and even value-tainted, the prospect of salvaging them for completely consensual, value-neutral use may be bleak. However, a much more promising enterprise may be to consider and categorize the different types of end-of-life practices, as this may better reflect the intricacy and complexity of end-of-life decision-making. The main previous work in this area that I am aware of is the “medical decisions concerning the end of life” (MDEL) system devised in the first Remmelink Report (van der Maas, van Delden, and Pijnenborg 1992), which was principally designed for conducting questionnaire-based empirical research into the end-of-life practices which were already happening in the Netherlands at the time, to inform subsequent policymaking.

The Remmelink investigators correctly acknowledge that “defining what is and is not euthanasia was not sufficient.” They also attempt, where possible, to use descriptive terms and avoid any value-loaded terms, although recognize that “it was nevertheless not possible to completely exclude morally charged terms in an investigation of this nature” (19). They approach their categorization system from the perspective of physicians, given that physicians were the ones answering most of the survey questions, whilst recognizing that “differences that are relevant for physicians may be of little relevance to legal experts and vice versa”; although this was balanced against the need to keep the categorization system suitable for drafting subsequent legislation (19). They also acknowledge that, irrespective of what approach is taken to categorization, “there will always remain problems of delineation, of borderline cases or situations” (19).

The Remmelink authors base their discussion around four questions (20):

1. What does the physician do?
2. What is the physician’s intention in doing this?
3. Did the patient request this intervention?
4. Was the patient able (or not) to decide upon this intervention?

Regarding their first question, the Remmelink authors state that the physician can either “administer drugs that (possibly) hasten the end of life, withhold or withdraw a (possibly) life prolonging treatment” (21). Regarding their second question, they state that the physician either (A) acts with the explicit intention of hastening the end of life, or (B) partly with the purpose of hastening the end of life, or (C) acting whilst taking into account the probability that the end of life will be hastened. Regarding scenario B above, they state that “sometimes an intervention is performed to achieve one particular effect (e.g. pain relief) but the side-effect (e.g. death) is not unwelcome. Strictly speaking, this situation should be categorized as intentional intervention” (21). This middle B category of acting “partly with the purpose” of hastening the end of life was included to reflect the “situation in which death of the patient was not foremost in the physician’s mind but neither was death unwelcome” (21). Regarding scenario C above, they state that “in order to be considered unintentional, this side-effect should in fact not have been desired” (21). Note that this sets potential grounds for ethical and legal evaluation appealing to Aquinas’s doctrine of double effect (see McIntyre 2019).

Regarding their third question, the Remmelink authors (1992) state that “the (explicit) request of the patient even forms part of the definition of euthanasia”; however, they distinguish the “explicit” and “permanent” request that is “adhered to voluntarily and after consideration,” from the less concrete request such as when “on hearing an ominous diagnosis, a patient can ask his physician if he would be prepared to terminate life if, in due course, suffering would become unbearable” (22). In this latter case, they explain that “the patient absolutely does not intend that any action should be undertaken at that moment and in a number of cases there will never be a request for direct intervention” (22); this was expressed in their questionnaire as a “request to perform euthanasia or assist with suicide in due course” (22). Regarding such requests in the “foreseeable future” (as opposed to in the present or at a specified future time point), they further separate “spontaneous” from “explicit and repeated” requests. They also specifically state that DNR orders constitute an MDEL (24).

Regarding their fourth question, the Remmelink authors distinguish patients who are able to make a decision for euthanasia from those who cannot, although they recognize difficulties in defining and determining decision-making ability. They state that the usual understanding of decision-making capacity is “being able to appreciate the nature of (assess) the situation so as to reach a decision adequately” (23), and this is importantly *irrespective of the outcome* of the actual decision; it refers only to the *process* of making said decision. They also allow for a middle situation for someone with “partial” decision-making capacity, thereby committing to a spectrum of decision-making capacity (rather than it being quantally present or absent).

In defining their MDELs, the Remmelink authors appreciate the vagueness of the concept of the “terminal phase” of an illness and instead avoid this terminology altogether and phrase their questionnaire according to a quantitative estimated prognosis. This was worded as how long “the life of the patient was in fact shortened by the action taken” (23), or in the case of withholding or withdrawing life-sustaining treatment, the decision to “not prolong life by a certain period of time rather than to shorten life by the same period of time” (23). Note that these are both counterfactual claims, comparing the difference between time X (when death occurs with the MDEL being carried out in this actual universe) and time Y (when death would otherwise have occurred “naturally” in another parallel universe if no MDEL was carried out). They acknowledge that many physicians were reluctant to answer these questions owing to significant epistemological challenges in prognostication, although they reason that “if, however, in a large number of cases the physicians indicated that life was shortened by a maximum of hours or days, the cautious assumption can be made that the patient was dying” (24). In contrast, they also reason that if “physicians indicated that life was shortened by weeks or months, one can assume that seriously ill patients were involved who were not yet dying” (24). Note that the Remmelink authors are using the phrase “terminal phase” as synonymous with the “dying phase” of an illness, i.e., the last hours to days of life, in which the body gradually and irreversibly shuts down, including neurological (level of alertness/arousal) and cardiopulmonary functions (breathing rate and pattern, oxygen levels, heart rate, blood pressure). This is separate from the concept of a “terminal illness” as used in this essay, which refers to the diagnosis itself (e.g., widely metastatic bowel cancer) without any reference made to the specific prognosis measured in hours to days.

### Tabulating the Logical Space of “End-Of-Life Practices”

Turning now to the second half of my thesis, I argue that the six disputed definitional questions identified in section 1 can be broken down into six “building blocks” and combined to map the logical space of end-of-life practices in this context, which I will call the taxonomy of “end-of-life practices” so as not to conflate them with the MDELs just discussed. Whilst covering *some* of the same ground as the MDEL system, the taxonomy is designed to exhaustively delineate each possible end-of-life practice including the categorization of borderline cases, for the purpose of aiding ethical arguments and policy making, as opposed to the MDEL’s primary purpose of optimizing the design of questionnaires used for large scale empirical studies which were subsequently translated into policy.

The six building blocks are as follows:

1. The type of practice (type-status): (i) killing, (ii) assisting suicide, or (iii) letting die (by WWIFLSTs).
2. The provider’s intention of hastening or bringing about death (intention-status): (i) fully/primarily intended, (ii) partially/secondarily intended (or not unwelcomed), or (iii) merely foreseen (but unintended).
3. The nature of patient’s request (voluntariness-status): (i) voluntary, (ii) nonvoluntary, or (iii) involuntary.
4. The patient’s medical condition-status: (i) terminally ill, or (ii) not terminally ill.
5. The patient’s consciousness-status: (i) fully conscious, (ii) in a permanent coma or PVS but has once been fully conscious, or (iii) has never been and never will be fully conscious (i.e., in newborns).
6. The patient’s suffering-status: (i) suffering, or (ii) not suffering.

For clarification, regarding the first building block, I have chosen to commit to the word “kill” (as a point of difference from Beauchamp and Davidson) because the alternative phrase “causing the death of” is ambiguous as to whether this denotes killing or the set containing both killing and letting die, dependent upon one’s theory of causation. I use the term “hastening or bringing about death” to denote this set and emphasize that “kill” is being used as a value-neutral placeholder, which could theoretically be substituted for another word or phrase if desired. Regarding the fifth building block, I am using the term “fully conscious” to mean “not in a permanent coma or PVS,” despite the fact that these “fully conscious” patients will of course be unconscious when they are asleep. By “has never been and never will be fully conscious” I am referring to newborns with severe congenital neurological defects resulting in them never being fully conscious, e.g., owing to anencephaly (being born without a major portion of the brain, skull, and scalp). The importance of this distinction from adults in permanent comas or PVSs will become clear shortly.

With a view to being as inclusive as possible, these six building blocks could be combined to delineate 3 × 3 × 3 × 2 × 3 × 2 = 324 different practices; however, many of them turn out to be nonsensical or impossible upon deeper consideration. Tabulating these combinations to determine which ones do and don’t plausibly exist is a lengthy but worthwhile task. To do this, the medical condition-status and consciousness-status are used to create four tables: table 1 pertains to fully conscious, terminally ill patients; table 2 to fully conscious, non-terminally ill patients; table 3 to patients in permanent comas or PVSs who were once fully conscious; and table 4 to patients who have never been and never will be fully conscious. The terminally ill/not terminally ill dichotomy loses most of its importance in patients in permanent comas or PVSs; hence, the medical condition-status does not apply to Tables 3 or 4, so we can get away with having four tables in total instead of six. In each table, the type-status forms the rows, the intention-status forms the columns, and the voluntariness-status is listed three times as sub-rows in each cell of the table. These tables therefore incorporate combinations of the first five criteria. This will be followed by a discussion of how the sixth building block relates to this schema; for the interim, every patient in each of the following four tables is considered to be suffering unless otherwise stated. To identify a specific end-of-life practice, choose the relevant table using the medical condition- and consciousness-statuses, and then triangulate it within that table using the type-, intention-, and voluntariness-statuses.

**Table 1:** In fully conscious, terminally ill patients

Type-status	Intention-status (provider’s intention of bringing about death)		
	Fully intended	Partially intended	Merely foreseen
Killing	Voluntary(^) Nonvoluntary Involuntary	Voluntary Nonvoluntary Involuntary	Voluntary Nonvoluntary Involuntary
Assisting suicide	Voluntary(^^)	Voluntary(*)	Voluntary
Letting die	Voluntary Nonvoluntary Involuntary	Voluntary Nonvoluntary Involuntary	Voluntary Nonvoluntary(**) Involuntary(***)

**Table 2:** In fully conscious, non-terminally ill patients

Type-status	Intention-status (provider’s intention of bringing about death)		
	Fully intended	Partially intended	Merely foreseen
Killing	Voluntary Nonvoluntary Involuntary		
Assisting suicide	Voluntary		
Letting die	Voluntary Nonvoluntary Involuntary		Voluntary Nonvoluntary Involuntary

**Table 3:** In patients in permanent comas or PVSs who were once fully conscious

Type-status	Intention-status (provider’s intention of bringing about death)		
	Fully intended	Partially intended	Merely foreseen
Killing	Voluntary(****) Nonvoluntary Involuntary		
Assisting suicide			
Letting die	Voluntary Nonvoluntary Involuntary(*****)		Voluntary Nonvoluntary Involuntary

**Table 4:** In patients who have never been and never will be fully conscious

Type-status	Intention-status (provider’s intention of bringing about death)		
	Fully intended	Partially intended	Merely foreseen
Killing	Nonvoluntary		
Assisting suicide			
Letting die	Nonvoluntary		Nonvoluntary

To explain how to read this first table, consider the practice with the (^) next to it. This practice is in table 1 so this means that the patient is fully conscious and terminally ill, e.g., with end-stage metastatic bowel cancer. It is in the “killing” row so the practice is a killing, e.g., the doctor administers the patient a lethal injection of pentobarbital. It is in the “fully intended” column so the doctor fully intends to kill the patient with the injection. Finally, it is in the “voluntary” sub-row so the patient provides informed consent to the lethal injection; he voluntarily requests it. This practice is the paradigm case of euthanasia offered initially. The practice with the (^^) next to it is the same except for that it is in the “assisting suicide” row, so rather than injecting the patient with pentobarbital, the doctor has given the patient a combination of lethal oral drugs for him to take himself at home. This practice is the paradigm case of assisted suicide offered initially.

Consider the practice with the (*) next to it. This practice is also in the “assisting suicide” row so it is an assisted suicide. It is in the “partially intended” column so the doctor only partially intends for his involvement to result in the patient’s death. This is only possible if the doctor’s involvement has another, primary intention. In this context that primary intention is probably pain relief. The doctor may have given the patient lots of analgesia, e.g., morphine, with the primary intention of providing sufficient analgesia to relieve the patient’s pain, although with the partial/secondary intention (or at least the not unwelcomed outcome) of either hurrying along the dying process, or alternatively, empowering the patient to commit suicide if he so chooses. The doctor carefully explains to the patient how much of the morphine he would need to take to commit suicide and then leaves the decision of how much to take up to him. Finally, the practice is in the “voluntary” sub-row so the patient has voluntarily requested to be assisted to suicide. Notice that there are no “nonvoluntary” or “involuntary” sub-rows in the “assisting suicide” row. These practices could only be possible if the patient is placed in a situation where he could “accidentally” commit suicide or is forced to commit suicide, respectively. These are too farfetched to warrant serious consideration, and on most accounts suicide cannot be accidental or involuntary by definition (Cholbi 2021).

Consider the practice with the (**) next to it. Unlike before, this practice is in the “letting die” row so the patient is allowed to die; in the medical context this is achieved by WWIFLSTs, e.g., through DNR orders. It is in the “merely foreseen” column so bringing about or hastening death is not intended; instead, the WWIFLSTs is intended as a direct means to relieving suffering (independent of death coming about) or as an end in itself.^5^ Finally, it is in the “nonvoluntary” sub-row so the patient does not provide informed consent to, or expressly not consent to, the letting die, e.g., because his decision-making capacity has been undermined by late-stage Alzheimer’s disease. Hence, the DNR order must have been implemented by a surrogate decision-maker, e.g., the patient’s family or medical team. Conversely, if this patient had instead written an ACD himself stating that he would not want resuscitation in the future (and provided that this ACD was written in the past at a time in the early stages of his disease when he still had adequate decision-making capacity), then this end-of-life practice would instead constitute the “voluntary” end-of-life practice immediately above the one marked (**). Interestingly, 80 per cent of DNR orders are implemented by surrogate decision-makers (Fins, McCarthy, and Limehouse 2017, 8).

Consider the practice with the (***) next to it. This is identical to the last practice except that it is in the “involuntary” sub-row: the patient has expressly not consented to being allowed to die. This would be the case if a medically competent, dying patient requests artificial nutrition and hydration but his medical team make a unilateral decision not to provide it to him on the grounds that it is inappropriate or futile.

It is also interesting to note that—in one study across six countries—45 per cent of letting die occurred with the full intention of hastening or bringing about death (Bosshard et al. 2006), unlike cases (**) and (***) just described.

Now, consider the second table:

This table is less populated than the first; some additional practices have been ruled out, not because they are impossible but because they are too farfetched to merit serious consideration. Firstly, consider partially intended or merely foreseen killing or assisting suicide. A patient who is not terminally ill can, by definition, possibly be cured. He might nonetheless be suffering owing to pain or other symptoms. Opioids or sedatives can be administered or provided to him in non-lethal doses. To administer or provide those drugs in lethal doses would suggest that hastening or bringing about death was intended to an extent that it could reasonably be considered the primary intention*.* Consider a patient with severe, debilitating arthritis. His pain can be substantially relieved through a non-lethal dose of opioids (or other analgesics). If a lethal dose is used, it would seem strange for the doctor to claim that he was primarily intending to relieve pain and that hastening or bringing about his patient’s death was only partially intended or merely foreseen. Given that death could have been avoided, and that death is usually a more significant outcome than relieving pain in patients who are not already dying, it can reasonably be claimed that the doctor’s actual primary intention was bringing about death.

However, consider merely foreseen letting die. When a patient is not terminally ill, cure is by definition possible, so medical intervention aimed at achieving a cure cannot be “futile” in the way that we have defined medical futility. Nonetheless, intervention in these patients can be considered “inappropriate” in three ways. Firstly, sometimes curative treatment is inappropriate because it is unwanted: consider a Jehovah’s Witness refusing a life-saving blood transfusion on religious grounds. In this case the doctor respects the Jehovah’s Witness’s decision and foresees that he will die, although certainly does not intend for him to die. I am aware that, strictly speaking, “life-saving” and “life-sustaining” treatments are not the same things: life-saving treatment is curative whereas life-sustaining treatment is not. However, life-saving treatment also sustains life, i.e., it allows life to continue. Hence, it is reasonable to consider life-saving treatment as a *subset* of (i.e., a form of) life-sustaining treatment, so conflating the two is acceptable here. Secondly, sometimes achieving a cure is *unlikely,* and aiming at that cure can produce more harm than good by prolonging suffering and delaying a likely death; intervention against these odds can sometimes be considered inappropriate. A difficult course of surgery, chemotherapy, and radiotherapy with only a 5 per cent chance of success might fall into this category provided that the patient does not request the treatment. Finally, curative treatment might be inappropriate on economic grounds. Unfortunately, demand for medical resources usually exceeds supply, so difficult decisions as to who will receive life-saving treatment and who will not are sometimes unavoidable. Suppose that two patients need a hospital bed with a mechanical ventilator but only one is available; without the bed both patients will die. It is inappropriate in one sense to provide the bed to the patient with the lower chance of being cured (call him “A”) than to the patient with the higher chance of being cured (call him “B”). This is the case irrespective of whether A consents to or expressly does not consent to B getting the bed or if A does not consent at all because he lacks the capacity to do so. For these reasons, merely foreseen letting die *is possible* in non-terminally ill patients irrespective of whether it is voluntary, nonvoluntary, or involuntary; avoiding inappropriate treatment is the sole end being aimed at.^6^

Finally, consider partially intended letting die. This is ruled out in non-terminally ill patients for the same reasons that partially intended killing and assisting suicide were ruled out. In patients who are not already dying, hastening or bringing about death can be more easily avoided than in patients who are dying, and in non-terminally ill patients death is usually a more significant outcome than avoiding inappropriate treatment. Therefore, if bringing about or hastening death is partially intended, then it can reasonably be claimed that it was actually fully intended, since otherwise the death could have been avoided altogether.

Now, consider the third table:

Whilst acknowledging the debate on the whole brain versus higher brain definition of death—and therefore whether or not patients in permanent comas or PVSs are actually living persons—suppose that they *are* living persons for the sake of taxonomic inclusiveness. Consider the practice with the (****) next to it. This practice is in table 3 because the patient is in a permanent coma or PVS but was once fully conscious. It is in the “killing” row because the patient is killed, e.g., the doctor administers the patient a lethal injection of pentobarbital. It is in the “fully intended” column because the doctor fully intends to kill the patient with the injection. Finally, it is in the “voluntary” sub-row because the patient provides informed consent to the lethal injection; he voluntarily requested it via an ACD written before he entered his permanent coma or PVS. It is contentious as to whether or not ACDs really are capable of providing informed consent to future medical decisions; again, suppose that they can for the sake of taxonomic inclusiveness.

Consider the practice with the (*****) next to it. Unlike before, this practice is in the “letting die” row because the patient is allowed to die by WWIFLSTs. It is in the “fully intended” column because the doctor fully intends for the WWIFLSTs to hasten or bring about the patient’s death. Finally, it is in the involuntary sub-row because the patient expressly did not consent to WWIFLSTs; he wrote in an ACD before he entered his permanent coma or PVS that he wanted to be kept alive for as long as possible. It should be noted that almost all interlocutors would argue that this does not entail any ethical obligation on the doctor to provide futile treatments (even if the patient has requested it), and that the doctor also has an ethical obligation not to inappropriately use scarce medical resources; however, these are now ethical evaluations which are separate to the value-neutral task of taxonomically defining each end-of-life practice.

The “assisting suicide” row is empty in table 3 because patients in permanent comas or PVSs cannot initiate voluntary movements; they physically cannot commit suicide.

Now, consider the fourth table:

The reasoning in table 3 applies to table 4 with one exception: patients who have never been and never will be fully conscious (i.e., infants born with severe neurological defects) could not have left ACDs and so cannot consent to, or expressly not consent to, future medical decisions. Hence, all decisions made must be nonvoluntary. A surrogate decision-maker (such as a parent) can make decisions on behalf of the patient that are deemed to be in the patient’s best interests, although the patient himself is not actually providing this informed consent so it is necessarily nonvoluntary from his perspective.

### Adding the Sixth Building Block

Now turning to the sixth suffering-status building block, in the context of end-of-life decision making most fully conscious patients will be suffering (either physically, or at least emotionally/psychologically or socially), irrespective of whether or not they are terminally ill. As discussed in section 1, many believe that any discussion surrounding euthanasia or assisted suicide in the absence of suffering is misplaced; however, we have also explored counterarguments to this claim, so for the sake of taxonomic inclusiveness this suffering versus not suffering dichotomy can be included. All of the practices in tables 1 and 2 can therefore be duplicated. For example, the first practice in table 1 discussed, marked with the (^) symbol, now becomes two practices: (^^A^) in a person who is suffering, and (^^B^) in a person who is not suffering. Regarding patients who are in permanent comas or PVSs, as discussed in section 1 it is contentious as to whether or not these patients are capable of experiencing suffering; if they can, then each practice in tables 3 and 4 is also duplicated, whereas if they cannot, then the practices in these tables are not duplicated. Note that I have not defined a middle “partially suffering” category owing to the metaphysical difficulty of drawing a non-arbitrary line on the spectrum of suffering, as discussed earlier.

### Overlapping Practices

To complicate matters, many of the practices delineated in these four tables are not mutually exclusive. WWIFLSTs, and the administration of opioids or sedatives, almost always occur in conjunction. It may be the case that one of the two brings about death, or both may be individually sufficient to bring about death (resulting in overdetermination), or both may be required to bring about death (thereby both “contributing to” or “hastening” death). Determining which of these is the case *in practice* is another problem altogether, which falls beyond the scope of this essay. However, this overlap is less problematic than it appears prima facie; in all of these cases, both practices at least “hasten” death, irrespective of whether or not they are necessary or sufficient to “bring it about.” Hence, both practices fall within the hybrid “hastens or brings about death” category used in the taxonomy.

Although it might seem that some aspects of a single end-of-life practice may occur concurrently and contradict each other, it is reasonable to claim that certain aspects of the practice trump each other. There cannot be a clash within the patient’s medical condition-status or consciousness-status; a patient cannot be terminally ill and not terminally ill at the same time or fully conscious or in a permanent coma or PVS at the same time. Similarly, there cannot be a clash within the suffering-status; a patient cannot be simultaneously suffering and not suffering. However, there could prima facie be a concurrent killing (e.g., via opioids and sedatives) and a letting die (via WWIFLSTs), the former of which might be “fully intended” and “involuntary,” and the latter of which might be “merely foreseen” and “voluntary.” But it would be nonsensical to claim that someone is killed and allowed to die at the same time, or that their hastening or bringing about of death is simultaneously fully intended and merely foreseen, or simultaneously voluntary and involuntary. Clearly “killing” trumps “allowing to die,” “fully intended” trumps “partially intended,” which in turn trumps “merely foreseen,” and “involuntary” trumps “nonvoluntary,” which in turn trumps “voluntary.” In this case, the patient is involuntarily killed with the full intention of killing him, rather than voluntarily being allowed to die with his death being a merely foreseen consequence of WWIFLSTs.

### Why the Value-Neutral Taxonomy Is Important and How It Can Be Utilized

With the view to inclusiveness that I have just adopted there are forty-three end-of-life practices in the four tables (accounting for criteria 1–5). If accounting for the sixth criterion, this number increases to eighty-six. A nifty abbreviation system acting as a set of coordinates could potentially be introduced to quickly identify each of the various practices, although I fear that it would add to the complexity rather than reduce it. Speaking in the specific terms of these end-of-life practices is cumbersome; no wonder interlocutors prefer to use names which lump some of them together. However, the fundamental problem is that different interlocutors group different combinations of these eighty-six practices together under the same names of “euthanasia” and “assisted suicide.” This may result in debaters unwittingly talking past each other, which is entirely counterproductive and potentially dangerous given the tangible impact that such decisions have on patients and their families. Neil et al. (2007) and van der Heide et al. (2003) recognize these dangers; accordingly, they deliberately avoid use of the terms “euthanasia” and “assisted suicide” altogether.

I acknowledge that the terms “killing” and ‘suicide” have negative connotations and might be criticized as reflecting an ethical bias. I want to stress that I really am only using these terms as value-neutral denotations. If this was considered an overwhelming concern, each of these contentious words could be substituted for other placeholders without affecting any of the arguments or conclusions laid forth in this essay. 

There are two approaches moving forward based on this taxonomy. The first is to take the most orthodox component of the six building blocks, and to define the corresponding single end-of-life practice as “euthanasia” (i.e., the paradigm case offered initially), or to take a few of the most orthodox components of the building blocks, and define the set of corresponding end-of-life practices as “euthanasia” (i.e., the paradigm case and other practices similar to it). The same strategy applies for “assisted suicide.” The second and probably more grounded approach—albeit not mutually exclusive with the first—is to be wary of and consider each of the practices individually, since it might be that two very similar practices (which may be lumped together under the same name of “euthanasia”) may differ in that one ought to be ethical and the other unethical, or that legislation ought to be written such that one is deemed legal and the other illegal. Otherwise, there is the most serious threat that a specific end-of-life practice may be unwittingly assigned an incorrect ethical and/or legal status. 

The complexity of this schema makes it inappropriate for daily clinical use by physicians on their ward rounds. However, I envisage that it may be utilized as a solid platform upon which substantive ethical and legal discussion—unscathed by ambiguous and value-loaded terms—can continue. I anticipate its usefulness at the meta- and normative-ethical levels. In particular, bioethical thought experimentation in the spirit of Foot, Thomson, Rachels, and Singer would be benefited when arguing for ethical connections and disconnections between the various delineated end-of-life practices. I also anticipate its usefulness as an aid for careful policymaking, along with benefiting the design of any future empirical studies which could then be more reliably interpreted and compared. If nothing else, I hope that the taxonomy at least promotes a greater appreciation of the range of different practices at the end of life, reveals some of the hidden metaphysical challenges underlying many of these distinctions, and ultimately offers a thorough way to think about the various potentially ethically and legally significant aspects of end-of-life decision-making.

## Data Availability

Not applicable.

## Footnotes

1 For the sake of consistency, I will use male characters and pronouns in this essay. Gender is completely irrelevant to all of my examples and to the taxonomy which is eventually developed.

2 Note that I have chosen to treat *withdrawing* treatment as an omission, and in doing so lump it together with *withholding* treatment, even though this metaphysical claim is contestable. There are arguments that withdrawing treatment actually constitutes an action, as well as arguments that the dichotomy between actions and omissions is flawed altogether (Harris 1975; Len Doyal 2006; Len Doyal and Lesley Doyal 2001; Warnock 1998; Woollard and Howard-Snyder 2021). In my opinion both of these lines of argument are strong and require consideration; however, for the purpose of developing the taxonomy which follows in this essay, I am treating both withdrawing treatment and withholding treatment as omissions, so as to accord with the orthodox use of the terms in the literature (American Academy of Hospice and Palliative Medicine AAHPM 2011; Fins, McCarthy, and Limehouse 2017; Jonsen, Siegler, and Winslade 2010; Karlawish and James 2009).

3 Note that if a patient is simply not asked whether or not he would like to be euthanized, despite him having the capacity and opportunity to answer this question, then according to these definitions the euthanasia is nonvoluntary. However, given how obviously paramount it is to ask this patient such a question, failing to do so would more practically classify the resulting euthanasia as involuntary.

4 Their definition in full reads: “In summary, we have argued in this section that the death of a human being, A, is an instance of euthanasia if and only if (1) A’s death is intended by at least one other human being, B, where B is either the cause of death or a causally relevant feature of the event resulting in death (whether by action or by omission); (2) there is either sufficient current evidence for B to believe that A is acutely suffering or irreversibly comatose, or there is sufficient current evidence related to A’s present condition such that one or more known causal laws supports B’s belief that A will be in a condition of acute suffering or irreversible comatoseness; (3) *(a)* B’s primary reason for intending A’s death is cessation of A’s (actual or predicted future) suffering or irreversible comatoseness, where B does not intend A’s death for a different primary reason, though there may be other relevant reasons, and *(b)* there is sufficient current evidence for either A or B that causal means to A’s death will not produce any more suffering than would be produced for A if B were not to intervene; (4) the causal means to the event of A’s death are chosen by A or B to be as painless as possible, unless either A or B has an overriding reason for a more painful causal means, where the reason for choosing the latter causal means does not conflict with the evidence in *3b;* (5) A is a nonfetal organism” (304).

5 It is worth noting that WWIFLSTs can also sometimes aim at relieving suffering *indirectly,* via the intermediary step of achieving death, but in this case the death could not have been merely foreseen in the first place; it must have been at least partially intended.

6 Although recall the grey area surrounding the definition of “terminal illness” encountered earlier: in some senses the Jehovah’s Witness, the patient with the 5 per cent prognosis despite aggressive treatment, and person B are actually terminally ill and are therefore not accounted for by table 2 at all.
